# The Case for Reforming Drug Naming: Should Brand Name Trademark Protections Expire upon Generic Entry?

**DOI:** 10.1371/journal.pmed.1001955

**Published:** 2016-02-09

**Authors:** Ameet Sarpatwari, Aaron S. Kesselheim

**Affiliations:** Program On Regulation, Therapeutics, And Law (PORTAL), Division of Pharmacoepidemiology and Pharmacoeconomics, Department of Medicine, Brigham and Women’s Hospital and Harvard Medical School, Boston, Massachusetts, United States of America

## Abstract

Ameet Sarpatwari and Aaron Kesselheim explore whether stripping branded drugs of trademark protection would improve the efficiency and fairness of health care.

**Summary Points**

- Innovator drugs are given their own brand name and a common generic name that is shared with products certified as bioequivalent by a regulatory authority.
- Characteristics—including names—that uniquely identify a seller’s product are entitled to legal protection against misappropriation.
- Well-controlled studies and decades of use reveal that generic drugs approved by the Food and Drug Administration and European Medicines Agency are interchangeable with their innovator counterparts.
- Widespread use of brand names persists within health care systems even after market exclusivity ends on those products, resulting in wasteful expenditures on products for which substantially cheaper, generic equivalents are available.
- Allowing generic products to share the brand names of their corresponding innovator products could help reduce health care spending but would not prevent manufacturers from promoting their products or physicians from ensuring manufacturer-specific dispensing.

Prozac, Lipitor, Viagra, and numerous other brand names for prescription drugs have entered the common vernacular in the United States (US). This is for good reason, since pharmaceutical manufacturers spend at least US$30 billion annually on marketing brand awareness to US physicians and patients [1]. Globally, the use of brand names in such advertising has persisted largely unquestioned as a means for manufacturers to distinguish innovator drugs from each other and from their generic equivalents. However, use of brand names also means that each new prescription drug automatically receives two names—its brand name and generic name—and perhaps even more, as brand names can vary from country to country (Prozac is also called Erocap, Lorien, Lovan, and Zactin outside the US). While prescription drug brand names can increase medication name recognition by patients and help differentiate products, they can also confuse patients and reduce appropriate use of generic drugs. Given increased pressure to reduce drug costs and use medicines safely and effectively, can the prescription drug naming system be improved?

## Trademarks

In most countries, sellers may obtain legal protection for characteristics (e.g., name, design, and color) that uniquely identify their products as a form of intellectual property. The scope of this protection, however, is variable. Under US law, such “trademarks” cannot have a utilitarian function [2]. In one notable case, for example, the Supreme Court held that a company was not entitled to intellectual property protection for the visible dual-spring design of its wind-resistant signs because it was essential for the intended product use—to prevent the signs from blowing over [3]. By contrast, the utilitarian function doctrine applies only to shapes in the European Union (EU) [4]. Thus, a finding that color consistency among multiple versions of the same drug improves medication adherence [5,6] could be grounds for denying legal protection for pill color in the US, but not in the EU; the same principle would also apply to prescription drug brand names were a functional effect of such names established.

## Prescription Drug Naming and Generic Drug Equivalence

The current convention of issuing innovator drugs a brand name and a generic name is now a firmly entrenched feature of the worldwide prescription drug market. This practice originated from heated debates over prescription drug naming in the mid-20th century, which centered on balancing the policy goals of incentivizing innovation and limiting monopolies, and was hardly a foregone conclusion. In the late 1950s, US Senator Estes Kefauver (D-Tennessee), chairman of the Subcommittee on Antitrust and Monopoly, initially sought to ban brand names outright [7]. In the face of stiff opposition from the pharmaceutical industry, however, he was forced to accept a compromise: manufacturers would be able to continue using brand names for their drugs, but the Food and Drug Administration (FDA) would have authority to issue generic names applicable to all products sharing the same active ingredients.

The FDA has since delegated this authority to the US Adopted Names (USAN) Council, which—like other national naming organizations and manufacturers themselves—can recommend generic names to the World Health Organization (WHO) International Nonproprietary Names program. In approving such names, the WHO emphasizes simplicity and meaning, requiring the use of certain prefixes and suffixes that reveal information regarding drugs’ chemical compositions (e.g., ertapenem and imipenem are both carbapenem derivatives) or pharmacological effects (e.g., azithromycin and streptomycin are both antibiotics).

The generic name took on greater importance in the US and other countries in the 1980s, when the generic drug industry expanded in the wake of new legislation and programs allowing drug regulators to approve generic drugs on the basis of bioequivalence. For a product to be approved as a generic drug, it now must not only possess an equal amount of the same active ingredients but also be proven to deliver these active ingredients to a target site at an equivalent rate. These products receive the same generic name as their innovator counterparts. Robust research and decades of safe and effective generic drug use have demonstrated the interchangeability of generic and innovator drugs, particularly those authorized by high-quality regulators like the FDA and the European Medicines Agency. Even for so-called narrow therapeutic index drugs, for which small changes in dose have clinically significant effects, well-controlled studies have found no meaningful differences between generic and innovator products [8,9]. Generic drugs help establish a competitive marketplace and are usually much cheaper than innovator drugs, with savings exceeding 90% when ten or more generic versions of the same product are available. This reduction in cost, when passed on to consumers, has been associated with improved medication adherence and health outcomes [10,11].

## Continued Utilization of Brand Names and Its Consequences

Though inexpensive generic drugs may become available after an innovator drug’s market exclusivity ends, widespread utilization of brand names persists during that time. Between 2004 and 2008, 38% (105 of 277) of US news articles on industry-sponsored studies referenced drugs solely by their brand name [12]. Many physicians—influenced by the media, marketing, and mentors [13], as well the relative complexity of generic names—continue to prescribe drugs using brand names even after the exclusivity period for innovator products has ceased. An analysis of 25,238 outpatient visits to 1,342 US physicians in 2003, for example, found a 79% median frequency of prescribing multisource drugs by brand names [14]. For drugs long off-patent, there was little evidence that prescribing by generic names had improved over the previous decade [15]. Similarly, low rates of prescribing of multisource drugs by generic name have been reported in France [16,17]. In England, by contrast, physicians have achieved an 80% generic prescribing rate in part through a capitated payment model [17], suggesting that reform is possible.

**Fig 1:**
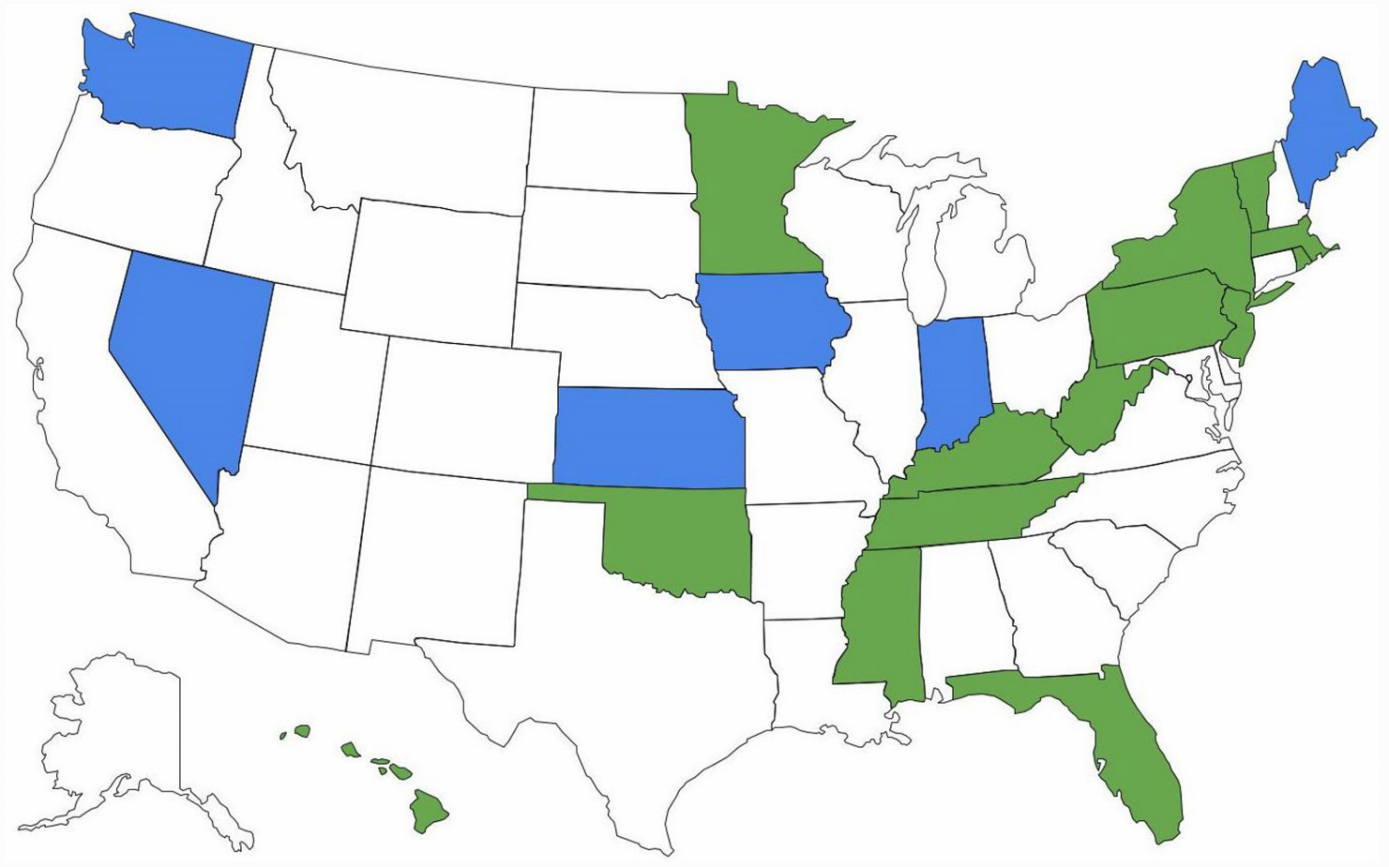
Pharmacists’ generic drug substitution obligations. Green = mandatory for all dispensings; blue = mandatory for dispensings paid for by government-sponsored insurance plans; and white = permissive.

**Fig 2:**
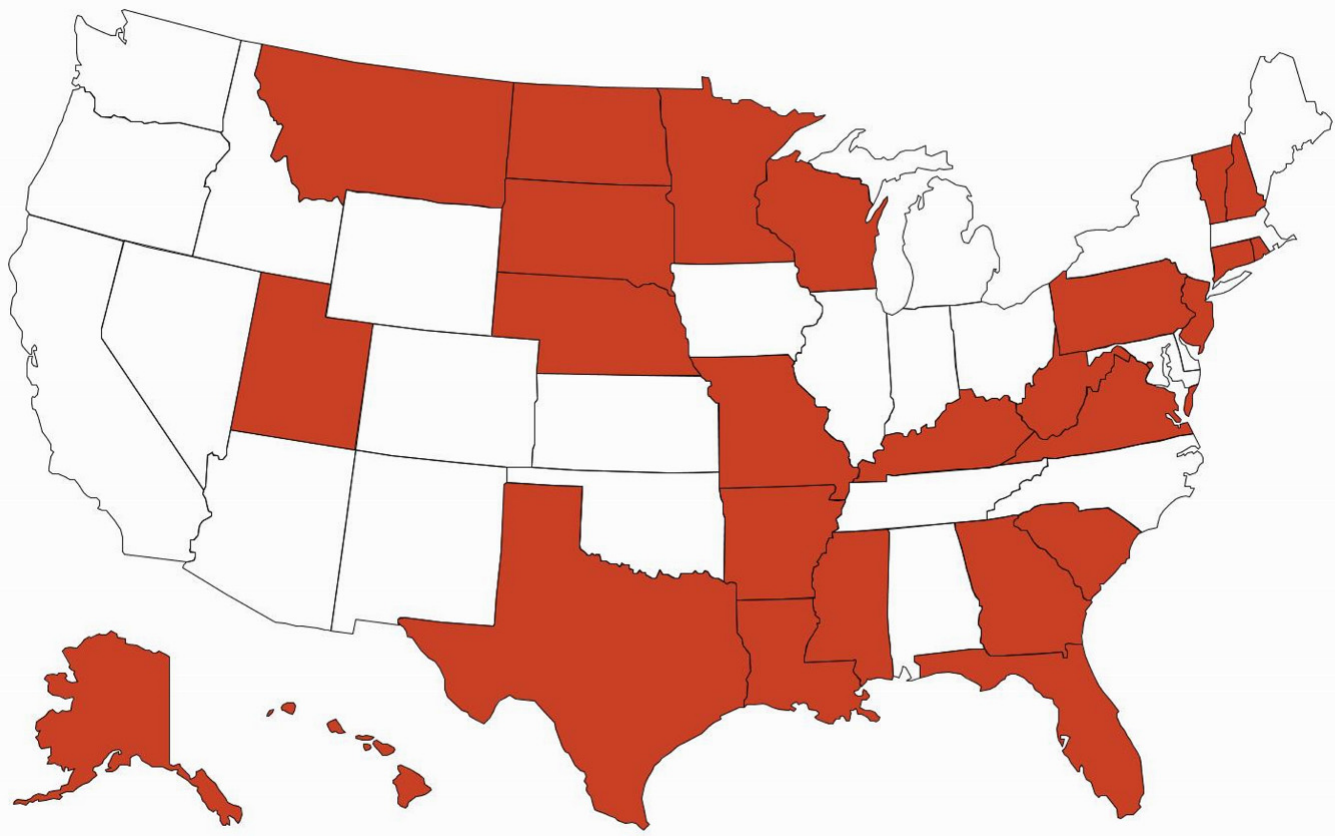
Patient consent for generic drug substitution. Red = required, and white = not required.

There are important ramifications of continued brand name use within health care systems. Of 15 EU countries evaluated by IMS Health in 2010, only seven (47%) had so-called drug product selection laws that authorize pharmacists to substitute prescriptions for innovator drugs with generic equivalents [18]. All US states have drug product selection laws, although they vary in important respects. Pharmacist-driven generic substitution is mandatory in only 20 states; by contrast, 26 states (including Washington, D.C.) require patient consent (Figs 1 and 2). The latter obligation was estimated to cost Medicaid, the combined state and federal health care safety net program for low-income patients, US$19.8 million in 2006 for the cholesterol-lowering drug simvastatin (Zocor) alone. Extrapolating this effect, investigators calculated that Medicaid would forgo US$100 million in potential savings in the year following the patent expiration of just three blockbuster drugs, atorvastatin (Lipitor), olanzapine (Zyprexa), and clopidogrel (Plavix) [19].

Pharmacist-driven generic substitution can additionally be circumvented by prescriptions for a drug’s brand name that the prescriber has specially marked with “dispense as written” or other similar sentiments. Of 5.6 million prescription claims submitted to a large pharmacy benefits manager in January 2009, 151,670 (2.7%) were designated as dispense as written, resulting in millions in forgone savings for the month [20]. Some US states have attempted to limit such prescriptions by requiring physicians to write out a specific phrase rather than checking a dispense-as-written box or prohibiting the use of prestamped dispense as written prescription pads, which have in the past been distributed by innovator manufacturers as promotional items.

Suboptimal generic substitution costs the US health care system US$12 billion annually [21]. In the EU, generic products account for just over 50% of all prescription drug dispensing [17]. These facts demonstrate that substantial savings remain possible internationally from greater generic drug use, which could be achieved by re-examination of laws and policies related to brand names.

## Possible Reform

One possible means of minimizing the negative impact of continued brand name use for prescription drugs would be to enact legislation permitting generic products to adopt the brand names of their corresponding innovator products. This policy would foster greater public confidence in the equivalence of generic and innovator drugs and help overcome some of the psychological and practical hurdles to generic substitution, resulting in substantial savings.

In evaluating the fairness of such a policy, an important consideration is the degree to which innovator drug manufacturers should be permitted to differentiate their products from generic equivalents. Such differentiation can drive innovator manufacturer profits. However, it also flies in the face of the compromise that produced the bioequivalence-based generic drug approval process—that generic products could be approved by the regulator using an abbreviated pathway based on pharmacodynamic comparability with innovator products in exchange for a defined period of market exclusivity for innovator manufacturers to profit.

The use of brand names by generic manufacturers, moreover, would limit—not foreclose—the ability of innovator drug manufacturers to distinguish their products in a multisource environment. Innovator manufacturers could still rely on their corporate name to establish a separate identity from generic drugs. Bayer, for example, lost its trademark for Aspirin, but still maintains a 25% share of the aspirin market in the US despite charging over three times the per-pill price of store-brand equivalents [22]. A physician who believes that the original version of a drug is indicated for a patient could in turn specify that manufacturer in a dispense as written prescription.

Some commentators have argued that drug product selection laws have already rendered trademark protections for brand names invalid in the period following market exclusivity of innovator drugs [23]. By authorizing pharmacists to substitute prescriptions for brand name products with generic equivalents, these laws have contributed to “genericide,” the evolution of a name from identifying a version of a product made by a particular manufacturer to “identifying the product itself” [23]. Thus, just as the names escalator, thermos, and zipper do not elicit a single company, so too can one argue that names like Prozac and Lipitor now encompass multisource products.

Allowing generic drug manufacturers to use brand names would offer several benefits over alternate policies to promote generic drug use. First, it would sidestep the convoluted nature of generic names, which could hamper efforts to increase generic drug prescribing. Although the era of intentional obfuscation by manufacturers is over, generic names like eslicarbazepine acetate and gadoterate meglumine remain exceedingly complex compared to their brand name counterparts Aptiom and Dotarem, in part owing to the admirable but misplaced effort by the WHO to select generic names that reveal information regarding drug structure or function (greater focus is instead needed on memorability and ease of use for the physician and patient). Co-option of brand names by generic manufacturers would also prove less costly than attempts to brand generics. Generic manufacturers would be able to capitalize on marketing undertaken by the innovator manufacturer rather than establishing an independent identity, enabling savings to be passed on directly to patients. Finally, brand name sharing would help mitigate the impact of cost-shifting tactics that have plagued insurer-led measures to incentivize generic drug use, such as patient assistance programs that cover the copayment cost premium of brand name products [24].

The continued use of two (or more) names for prescription drugs can cause confusion in the market and decreases the utilization of safe, effective, and more affordable generic products. Enabling generic products to adopt the brand names of their innovator counterparts would help reduce this inefficiency while still permitting product promotion and manufacturer-specific dispensing.

## Abbreviations

EU: European Union
FDA: Food and Drug Administration
US: United States
USAN: United States Approved Names
WHO: World Health Organization
